# A pilot trial of large versus small diameter needles for oocyte retrieval

**DOI:** 10.1186/1477-7827-11-22

**Published:** 2013-03-20

**Authors:** Vitaly A Kushnir, Ann Kim, Norbert Gleicher, David H Barad

**Affiliations:** 1 Center for Human Reproduction, 21 East 69th Street, New York, NY 10021, USA; 2 Foundation for Reproductive Medicine, New York, NY 10021, USA

**Keywords:** Oocyte retrieval, Needle, Ultrasound guided, Assisted reproduction

## Abstract

**Background:**

This study was designed to determine whether small diameter needles for oocyte retrieval alter oocyte yields in patients undergoing IVF in comparison to standard large diameter needles.

**Methods:**

We conducted a prospective pilot study of 21 consecutive favorable prognosis patients. In each patient one ovary was randomly allocated to retrieval with either a 20 G/ 35 mm (thin) or 17 G/ 35 mm (standard) needle, the other ovary was then retrieved with the opposite needle.

**Results:**

The standard diameter needle was used to collect a total of 215 oocytes from 355 aspirated follicles (60.6%) compared to 203 oocytes from 352 aspirated follicles (57.7%) with the thinner needle (p = 0.23). Stratifying outcomes by anti-Müllerian hormone (AMH), as indicator of ovarian reserve, and by body mass index (BMI) the oocyte yields, still, did not differ (AMH, r (17) = −0.20, p = 0.44; BMI, r (17) =0.02, p = 0.96). Outcomes also did not vary among women with diminished ovarian reserve (p = 0.17) and in women with normal ovarian reserve (p = 1.00). Operating time was, however, significantly increased by 3.3 minutes per ovary (z = −3.08, p = 0.002) with the thinner needle.

**Conclusions:**

Needle diameter does not affect oocyte yield, including in obese patients and patients with diminished ovarian reserve. Thinner needles appear to significantly prolong operating time.

## Background

Total number of oocytes retrieved is one of the most important prognostic factors in in vitro fertilization (IVF). Live birth rates following IVF increase with increasing number of retrieved oocytes up to about 15 [1]. With the recent emergence of in vitro maturation (IVM), even increased numbers of immature oocytes have the potential to improve chances of achieving pregnancy in an IVF cycle. Especially in patients with diminished ovarian reserve (DOR) or premature ovarian aging (POA), even a small increase in the number of oocytes has the potential to significantly improve pregnancy rates [2].

Few prospective studies have attempted to optimize oocyte yields during IVF [3-8]. Use of double lumen needles, allowing for improved flushing of follicles has not yielded improvements in number of oocytes compared to use of a single lumen needles [3-5,9]. Follicle curetting has been shown to improve oocyte yields, but not the pregnancy rate [6]. Decreasing needle size diameters from 15 to 17 or 18 gauge (G) has previously been shown to decrease pain, without affecting number of oocytes, their quality, or clinical pregnancy rates [7].

In this prospective study we, therefore, tested the hypothesis that a small diameter (20 G/ 35 mm) needle, primarily utilized in IVM cycles, compared to standard larger diameter (17 G/ 35 mm) needle, will result in more oocytes retrieved, especially from smaller follicles, containing potentially immature oocytes. A potential secondary goal was to determine whether selected patient characteristics, like obesity and DOR, might especially benefit from lower diameter needles.

## Methods

Since this study involved standard retrieval techniques and approved standard needles, in routine use at the center, the study underwent expedited approval by the center’s IRB. The patient population consisted of 21 consecutive favorable prognosis women undergoing routine IVF cycles at the Center for Human Reproduction in New York City. A patient was considered “favorable” if prior to retrieval she demonstrated two normal ovaries, with a minimum of six follicles per ovary on ultrasound, and estradiol greater than 2000 pg/mL on day of human chorionic gonadotropin (hCG) trigger, without exogenous estradiol support.

All endocrine measurements, including follicle-stimulating hormone (FSH) and anti-Müllerian hormone (AMH) levels, were obtained within six months of cycle initiation. Previously defined age-specific AMH values were used to differentiate women with normal (n = 15) and diminished ovarian reserve (n = 6) [10]. Ovarian stimulation was achieved with two stimulation protocols, women with normal ovarian reserve were down-regulated with GnRH-a (Lupron; TAP Pharmaceuticals, Deerfield, IL) agonist and stimulated with maximally 300 IU of gonadotropins daily; women with diminished ovarian reserve underwent microdose GnRH-a flare protocol followed by stimulation with 450 to 600 IU of exogenous gonadotropins daily. When ultrasound criteria for follicular maturity were met, a single 10,000 IU dose of hCG was administered. Transvaginal follicular aspiration was performed approximately 36 hours after hCG administration.

Three participating surgeons used, at random, first either the small diameter (20 G/ 35 mm Cook Medical, Bloomington, IN) or standard diameter (17 G/ 35 mm Rocket Medical, Hingham, MA) needle on the right ovary and then the other-size needle on the left ovary. Randomization was by coin toss at start of retrieval, performed by a staff person. Equivalent aspiration pressure of 200 mm Hg was used for all oocyte retrievals provided via Craft Suction Pump (Rocket Medical, Hingham, MA). Main outcome was number of oocytes retrieved per aspirated follicle.

### Statistical analysis

The study was powered to detect a 30% difference in number of oocytes retrieved per aspirated follicle with 80% power and 5% error. We estimated, based on historical data, an average of 14 oocytes (7 per ovary) in a typical favorable prognosis patient. The Wilcoxon Matched-Pairs Signed Ranks test, and correlation coefficients, were utilized where appropriate, with statistical software package, SPSS for Windows, version 18 (Chicago, Illinois). All tests were 2-tailed, with P < 0.05 significant. Values are reported as mean ± SD or median ± SEM as appropriate.

## Results

**Figure 1:**
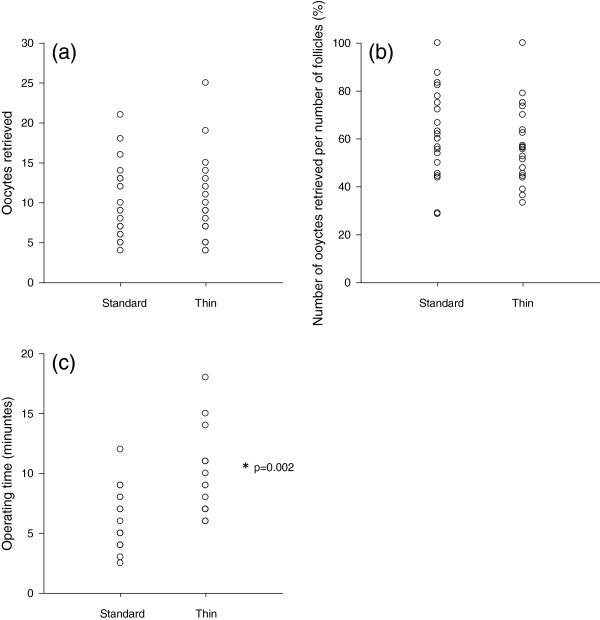
**Plots for standard (17 Gauge) needle and thin (20 Gauge) needle and cycle data. (a)** Oocyte yield did not differ between the two needles (p = 0.50). **(b)** The number of oocytes per the number of aspirated follicles did not differ between the standard and thin needles (p = 0.23). **(c)** Operating time significantly increased, by an average of 3.3 minutes per ovary (p = 0.002) using the thin needle in comparison to the standard needle. The asterisk indicates statistical significance (Wilcoxon Matched-Pairs Signed Ranks test).

Patients’ characteristic and cycle data are summarized in Table 1. Wilcoxon Matched-Pairs Signed Ranks test demonstrated that oocyte yields did not differ between the two needles (z = −0.67, p = 0.50; Figure 1a). The larger, standard diameter 17G needle was used to collect a total of 215 oocytes from 355 aspirated follicles (60.6%) compared to 203 oocytes from 352 aspirated follicles (57.7%) with the thinner, low diameter 20 G needle (z = −1.21, p = 0.23; Figure 1b). Operating time was, however, significantly increased by 3.3 minutes per ovary (z = −3.08, p = 0.002) with the thinner 20 G needle (Figure 1c).

**Table 1:** Patients’ characteristic and cycle data

**Number of patients**	**21**
Age (years)	30.7 ± 6.3
BMI (kg/m^2^)	23.6 ± 4.6
AMH (ng/mL)	3.6 ± 3.4
FSH (mIU/mL)	6.6 ± 3.0
Total gonadotropin dose (IU)	3600 ± 2028
**Standard (17 gauge) needle**	
Number of follicles	16.0 ± 1.4
Number of oocytes retrieved	9.0 ± 1.0
Operating time per ovary (min)	6.1 ± 2.5
**Thin (20 gauge) needle**	
Number of follicles	16.0 ± 1.7
Oocytes retrieved	9.0 ± 1.2
Operating time per ovary (min)	9.4 ± 3.3

Values shown are mean ± SD or median ± SEM as appropriate.

Stratifying outcome data by AMH levels of patients, as indicators of ovarian reserve, and by BMI there was, still, no difference observed in oocyte yields (AMH, r (17) = −0.20, p = 0.44; BMI, r (17) =0.02, p = 0.96).

**Figure 2:**
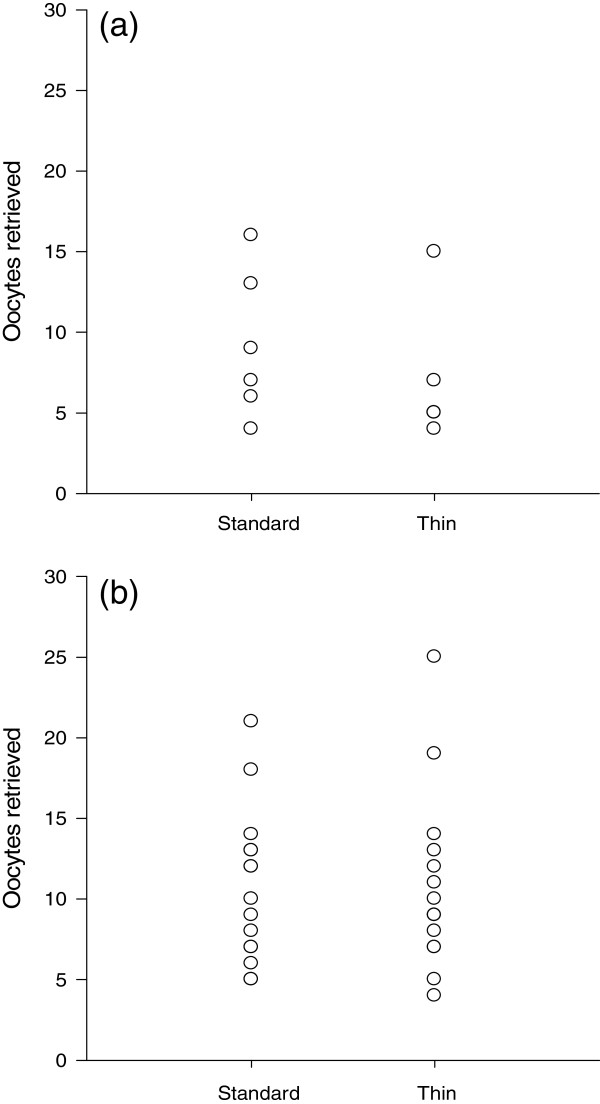
**Plots for needle size (Standard 17 Gauge and Thin 20 Gauge), ovarian reserve (diminished and normal), and oocytes retrieved. (a)** Oocytes yield did not differ between the two needles among women with diminished ovarian reserve (p = 0.17). **(b)** Oocyte yield did not differ between the two needles among women with normal ovarian reserve (p = 1.00). No statistically significant differences were observed (Wilcoxon Matched-Pairs Signed Ranks test).

Total gonadotropin dose was not significantly associated with BMI (r (17) = 0.43, p = 0.09); however, it was inversely associated with AMH (r (20) = −0.71, p < 0.001). AMH was inversely correlated with age (r (21) = −0.68, p = 0.001). As indicated by the Wilcoxon Matched-Pairs Signed Ranks test, standard (M = 9.2, SD = 4.5) and thin (M = 6.8, SD = 4.1) needles resulted in similar oocyte yields among women with diminished ovarian reserve (p = 0.17). Likewise, among women with normal ovarian reserve, oocyte yield did not differ between standard (M = 10.7, SD = 4.7) and thin (M = 10.8, SD = 5.4) needles (p = 1.00; Figure 2a,b). There were no adverse events observed during the study.

## Discussion

Since the introduction of sonographically guided vaginal oocyte retrieval [11], a surprisingly small number of studies have attempted to optimize the technique. The introduction of thin needles, primarily designed for in-vitro maturation (IVM), offers an opportunity to revisit the question of whether oocyte retrieval can be improved, giving better oocyte yields, possibly by retrieving oocytes from small follicles, which may not be retrievable by standard size needles. To test this hypothesis, we investigated a group of favorable prognosis patients under the assumptions that good prognosis patients should demonstrate any positive effect best because they are expected to produce highest oocyte yields.

Somewhat disappointingly, our data demonstrate that needle diameter does not seem to affect oocyte yield. Likewise, needle diameter does not seem to affect oocyte yield in obese patients or in those with diminished ovarian reserve. Since ovarian reserve does not affect outcome in good prognosis patients, it appears highly unlikely that a beneficial effect will be obtained in poor prognosis patients, though such a possibility cannot be completely ruled out, especially if only small follicles are present at time of retrieval. In women with severe DOR, even retrieval of a single immature oocyte can mean the difference between no pregnancy chance and at least a small chance of conception, as we have observed outside of this study in a number of cases (unpublished observations).

The study also demonstrates that routine use of smaller diameter needles significantly prolongs operative time. As expected, the flow rate was noted to be slower with the thin needle while using an equivalent aspiration pressure; this accounted for longer time to empty each punctured follicle. Spending more than three extra minutes per ovary in retrieval, when the small diameter needle is utilized, represents a significant disadvantage. While decreasing needle diameters has previously been shown to reduce post-operative pain [7,8] increased operative time may potentially increase complications.

While not formally studied, it was noted by all three surgeons that the small diameter needle encountered less tissue resistance and tenting, easing penetration of ovaries and individual follicles. This gives slightly more control to the surgeon, which may represent a slight safety advantage. The size of here reported study, however, allows no data-driven comment on safety differences between the two tested needle sizes.

Our studies main strength is the design which allows each patient to act as her own control; one ovary was retrieved with the small diameter needle while the contralateral ovary was retrieved with the large diameter needle. The main limitation of our study is that our patient population consisted of favorable prognosis patients; therefore, our findings cannot be necessarily, extrapolated to a more adversely selected patient population with diminished ovarian reserve. Post-operative pain and blood loss were not evaluated in this study since these parameters have been found to decrease with decreasing needle diameter [7,8,12]. A larger randomized study including poor prognosis patients would be helpful to determine whether a specific subgroup of patients would benefit from routine use of the small diameter needles.

Given all of here reported data and considerations, our center’s practice to use a larger size needle in routine oocyte retrievals has not changed. In women with very small follicle numbers, especially if follicles have to be retrieved at very small sizes, our center now, however, switches to a smaller diameter needle for retrievals of follicles under 10–12 mm size.

## Conclusions

Needle diameter does not affect oocyte yield, including in obese patients and patients with diminished ovarian reserve. Thinner needles appear to significantly prolong operating time.

## Competing interests

N.G. and D.H.B. are listed as co-owners of a number of already awarded and still pending U.S. patents, none related to the topic of this manuscript. N.G. is a shareholder in Fertility Nutraceuticals LLC and owner of the Center for Human Reproduction (CHR), where this study was conducted. N.G. and D.H.B receive patent royalties from Fertility Nutraceuticals, LLC.

## Authors’ contributions

VAK, NG and DHB were responsible for study design and acquisition of data. VAK and AK performed the analysis and interpretation of data; VAK wrote the manuscript. All authors read and approved the final manuscript.
